# Metagenomic Analysis of the Effects of *Lactiplantibacillus plantarum* and Fructooligosaccharides (FOS) on the Fecal Microbiota Structure in Mice

**DOI:** 10.3390/foods11091187

**Published:** 2022-04-19

**Authors:** Shumao Cui, Weiling Guo, Cailing Chen, Xin Tang, Jianxin Zhao, Bingyong Mao, Hao Zhang

**Affiliations:** 1 State Key Laboratory of Food Science and Technology, Jiangnan University, Wuxi 214122, China; cuishumao@jiangnan.edu.cn (S.C.); 7200112062@stu.jiangnan.edu.cn (W.G.); cailing0903@foxmail.com (C.C.); xintang@jiangnan.edu.cn (X.T.); zhaojianxin@jiangnan.edu.cn (J.Z.); zhanghao61@jiangnan.edu.cn (H.Z.); 2 School of Food Science and Technology, Jiangnan University, Wuxi 214122, China; 3 National Engineering Research Center for Functional Food, Jiangnan University, Wuxi 214122, China

**Keywords:** *Lactiplantibacillus plantarum*, fructooligosaccharide, short-chain fatty acids, intestinal microbiota

## Abstract

Understanding the association between food composition and intestinal microbiota in the context of individual health is a critical problem in personalized nutrition. The objective of the present research was to elucidate the influence of *Lactiplantibacillus plantarum* ST-III and fructooligosaccharides (FOS) on the intestinal microbiota structure. We found that *L. plantarum* ST-III and FOS interventions remarkably enhanced the levels of cecal short-chain fatty acids (SCFAs), especially acetic, butyric, and valeric acids. Moreover, *L. plantarum* ST-III and/or FOS intervention obviously altered the intestinal microbiota structure. At the genus level, *L. plantarum* ST-III and/or FOS intervention remarkably elevated the proportion of *Sutterella*, *Pediococcus*, *Proteus*, *Parabacteroides*, *Prevotella* and *Desulfovibrio*. Correlation analysis further uncovered that the specific compositional features of intestinal microbiota were strongly related to the concentration of cecal SCFAs. Our results offered scientific evidence to understanding the association between food composition and intestinal microbiota.

## 1. Introduction

The human gastrointestinal tract is regarded as a huge and complex ecology that colonizes trillions of microorganisms, which is composed of bacteria, fungi, protozoa, and so on. Over the past decades, the intestinal microbiota has attracted extensive attention worldwide, because it is an important contributor to multiple processes in human health and disease. Homeostasis of intestinal microbiota takes an essential role in facilitating the metabolism of nutrients, regulating immunity, controlling the glycolipid metabolism, reducing the permeability of the intestinal barrier, and effecting the susceptibility to numerous diseases [1]. In contrast, perturbations in intestinal microbiota balance are related to the process of obesity, diabetes, hepatitis, irritable bowel syndrome and other metabolic diseases [2]. Previous reports have shown that there are obvious differences in the intestinal microbiota structure between healthy volunteers and obese patients. For instance, patients with obesity show the increases of Firmicutes/Bacteroidetes ratio, and the decrease of proportion of *Akkermansia muciniphila* and *Lactiplantibacillus plantarum* [3]. In addition, intestinal microbiota takes an essential role in the conversion of food ingredients into small molecules, including short-chain fatty acids (SCFAs), tryptophan, vitamins B3, secondary bile acids and indole-3-propionate [4]. These metabolites are the focus of intense research, due to it exerting important and multiple effects on host physiology, especially SCFAs, which are saturated fatty acids with 6 carbon atoms that come from the fermentation process of undigested food by the microbiota in the gastrointestinal tract, which is strongly associated with the immune and related inflammatory responses. Previous study exhibited that diet containing fructooligosaccharides (FOS) significantly increased the cecal SCFAs concentrations, which helped improve the tyrosine metabolism pathway [5]. In addition, FOS supplementation remarkably elevated the proportion of *Bifidobacterium* and *Lactobacillus* [6]. *Bifidobacterium* and *Lactobacillus* belong to lactic acid bacteria (LAB), and are the most popular probiotics applied in the field of food and medicine. However, whether the presence of FOS interferes with the modulation of intestinal flora by *Lactobacillus* in vivo has been unclear.

Probiotics are considered as viable microbes, and induce health benefits to the host when supplemented in enough quantities (more than 10^9^ CFU). Probiotics prevent the intestinal microbiota disturbance, influence intestinal villi, and improve nutrient digestion and absorption. LAB are important probiotics and are commonly consumed by several healthy individuals. So far, certain LAB strains have been universally separated from the conventional fermented food products. Recent reports have elaborated that LAB strains take a vital role in shifting the intestinal microbiota composition, improving glycolipid metabolism, decreasing the risk of cardiovascular diseases, and modulating immune response [7]. In particular, *L. plantarum* exhibits the outstanding regulation of intestinal microbiota composition. For instance, *L. plantarum* intervention elevates the α-diversity of the intestinal microbiota, and enhances the proportion of *Adlercreutzia*, *Ruminococcus*, *Clostridium*, *Blautia* and *Akkermansia* in hypercholesterolemic mice induced by a high-cholesterol diet [8]. In addition, previous study showed that *L. plantarum* ST-III was involved in the metabolism of FOS by releasing β-fructofuranosidase [9]. Thus, elevating the relative abundance of *L. plantarum* helped to ameliorate host energy metabolism and regulate FOS metabolism. However, there are clear differences in the regulatory effect of LAB on intestinal microbiota composition, the usage of different LAB strains and dietary habits may be the major factor for the controversial results of LAB intervention [10]. Therefore, it is necessary to further resolve the controversy regarding the differential regulatory effect of LAB strains and dietary habits on the intestinal microbiota structure.

The objective of the present research was to estimate the influences of *L. plantarum* ST-III intervention on the gut microbiota structure in FOS or standard diet-fed mice. In addition, the potential correlation between gut microbial phylotypes and cecal SCFAs was revealed by correlation network analysis based on Spearman’s rank correlation coefficient. Our results offer the theoretical basis for the influences of *L. plantarum* intervention on the regulation of intestinal microbiota in mouse fed with different dietary habits.

## 2. Materials and Methods

### 2.1. Chemicals Reagents, and Preparation of L. plantarum ST-III

FOS consists of fructofuranosylnystose, nistose, and 1-kestose, and is offered by Baolingbao Biology Co., Ltd. (Dezhou, Shandong, China).

*L. plantarum* ST-III was collected from the fermented cabbage and cultured in MRS at 37 °C for 24 h. *L. plantarum* ST-III cultures were centrifuged (8000× *g*, 20 min, 4 °C), and washed 3 times with PBS. Then, the sediments were collected and were resuspended in PBS. The concentration of *L. plantarum* ST-III was approximately 1.1 × 10^10^ CFU/mL and stored at 4 °C until used.

### 2.2. Animal Design

Twenty-four C57BL/6N mice (♂, 7 weeks old) were obtained from SLAC Laboratory Animal Co. Ltd. (Hangzhou, China) and kept in the controllable environment (temperature: 23 ± 1 °C; relative humidity: 50 ± 5%; light/dark cycle: 12 h). The procedures of animal experiment were approved by the Ethics Committee of Jiangnan University on 15 June 2018 (JN. No20180615c2121101[104]).

After 7 days of acclimatization, all mouses were separated into 4 groups (*n* = 6), as follows: (1) control group: mice were fed a standard diet along with the oral administration of 0.2 mL PBS for 4 weeks; (2) FOS group: mice were fed an FOS diet along with gavage administration of 0.2 mL of PBS for 4 weeks; (3) Lp group: mice were fed a standard diet along with oral administration of 0.2 mL *L. plantarum* ST-III (2.2 × 10^9^ CFU) for 4 weeks; (4) FOS + Lp group: mice were fed an FOS diet along with oral administration of 0.2 mL of *L. plantarum* ST-III (2.2 × 10^9^ CFU) for 4 weeks. The detailed composition of the standard and FOS diet are shown in Table S1. The daily food intake and body weight were recorded. All mice were sacrificed after fasting for 12 h; the ileum, cecum and colon were obtained and weighed.

### 2.3. SCFAs Analysis

The cecal contents were decollated and stored at −80 °C. Cecal SCFA concentrations were detected following the approach described in our previous study [11]; 0.05 g of cecal contents and 0.5 mL of saturated NaCl solution were mixed and stored in 25 °C. After 30 min of static environment, the samples were mixed and homogenized at room temperature. The sample was acidified using 20 μL of 10% H_2_SO_4_ solution (*v*/*v*). Subsequently, 1 mL C_2_H_5_OC_2_H_5_ and solution were mixed. After the centrifugation (14,000× *g*, 20 min, 4 °C), the supernatants were collected and transferred to new centrifuge tubes (containing anhydrous Na_2_SO_4_), in order to adsorb the water in the solution. After centrifugation (14,000× *g*, 20 min, 0 °C), the supernatants were transferred to autosampler vials, and then the SCFAs levels were measured by gas chromatography-mass spectrometry (Thermo Fisher Scientific, Darmstadt, Germany).

### 2.4. DNA Extraction and Sequencing

The feces of mice in all groups were collected at 0 and 4 weeks. Bacterial DNA was extracted from the feces by a commercial kit (Qiagen, Hilden, Germany), following the manufacturer’s instructions. The levels of DNA were determined using a Nanodrop (Thermo Scientific, Waltham, MA, USA). Then, 1 μL of DNA was amplified using the primers (338F and 806R). The purity of PCR production was carried out using 1.5% agarose gel electrophoresis, and the concentrations of the PCR products were determined using a Nanodrop. Each sample was mixed according to the determined concentration, and the productions were carried out on a MiSeq platform (Illumina, San Diego, CA, USA).

### 2.5. Bioinformatics Analysis

All raw data were carried out using QIIME 2, and data with less than 97% similarity of operational taxonomic units (OTUs) were removed. The OTUs were annotated according to the SSUrRNA database. The α-diversity and proportions of intestinal microbiota were analyzed using MicrobiomeAnalyst (https://www.microbiomeanalyst.ca/, accessed on 17 April 2022). Principal coordinates analysis (PCA) of the intestinal microbiota was carried out online (http://www.cloud.biomicroclass.com, accessed on 17 April 2022). The association between the abundances of intestinal microbial genera and cecal SCFAs was carried out using R (v4.1.2) and Cytoscape (v3.6.1).

### 2.6. Statistical Analysis

All data are described as the mean ± SD using GraphPad Prism (v9.0). Significant discrepancies among all groups were calculated using SPSS software (v23.0). *p* < 0.05 are considered as statistically discrepant.

## 3. Results

### 3.1. Influence of L. plantarum ST-III and FOS Intervention on Body Weight and Organ Index

**Figure 1:**
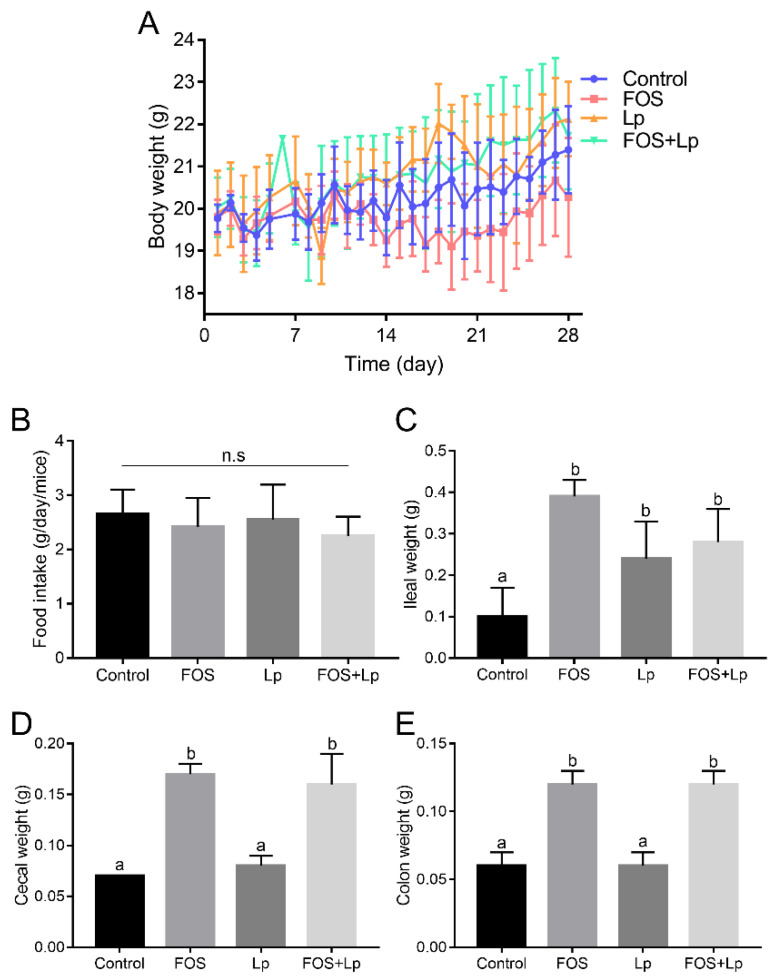
Influence of *L. plantarum* ST-III and FOS intervention on body weight (**A**), food intake (**B**), ileal weight (**C**), cecal weight (**D**), and colon weight (**E**). Different letters suggest a remarkable discrepancy according to *p* value (*p* < 0.05) between all groups.

Body weights of each mouse in the different groups were recorded during the experiment and are shown in Figure 1A. In the 1st week of the experiment, there were no obvious discrepancies in the body weight among all groups. From the 2nd week to the 4th week, long-time consumption of FOS markedly reduced the body weight relative to the control group (*p* < 0.05). However, *L. plantarum* ST-III intervention relieved the loss of body weight in FOS-fed mice. Interestingly, there were no obvious discrepancies in body weight between the control and Lp groups. Furthermore, during the experimental period, no obvious changes were observed in the daily food intake of mice among all the groups (*p* > 0.05) (Figure 1B).

The influences of *L. plantarum* ST-III and FOS intervention on the weights of the ileum, cecum and colon were explored. The ileal weight in the control group was lower than that of other groups (*p* < 0.05) (Figure 1C). Among these, the ileal weight of mice in the FOS group was higher than that in the Lp and FOS + Lp groups, and no obvious discrepancies were observed between the Lp and FOS + Lp groups. Interestingly, long-time consumption of FOS significantly increased the cecal and colon weight compared to that of mice fed with a standard diet (*p* < 0.05) (Figure 1D,E). Nevertheless, *L. plantarum* ST-III intervention did not change the cecal and colon weight in standard diet- or FOS-fed mice (*p* > 0.05).

### 3.2. Influence of L. plantarum ST-III and FOS Intervention on SCFAs

Cecal SCFAs are volatile fatty acids produced via fermentation by intestinal microbiota. Oral administration of *L. plantarum* ST-III slightly elevated the concentrations of SCFAs in mice fed-with a standard diet relative to the Control group, including that of acetic, butyric, valeric, and isovaleric acids, but no statistical differences between the Control and Lp group (*p* > 0.05) (Table 1). Interestingly, mice fed with FOS had significantly higher levels of SCFAs (containing acetic, propionic, butyric, valeric, and isovaleric acids) than mice fed with a standard diet (*p* < 0.05). In addition, the influence of *L. plantarum* ST-III intervention on SCFAs in FOS-fed mice was further explored. Apart from butyric acid, *L. plantarum* ST-III intervention slightly reduced the concentrations of SCFAs compared with FOS group (*p* > 0.05).

### 3.3. Alpha and Beta Diversity of Intestinal Microbiota

**Figure 2:**
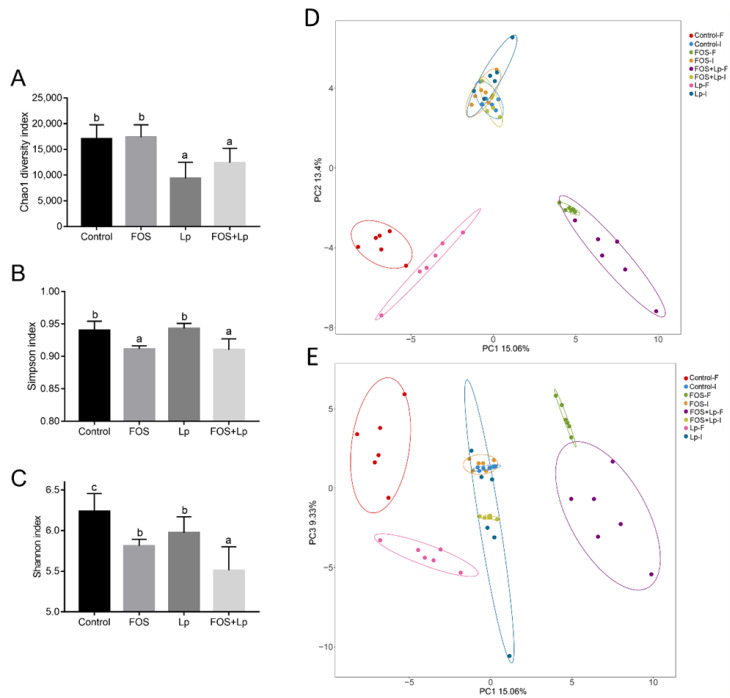
Influence of *L. plantarum* ST-III and FOS intervention on the gut microbiota. Chao1 index (**A**), Simpson index (**B**), Shannon index (**C**), PCA score plots (PC1*PC2, PC1*PC3), Control-I, FOS-I, FOS + Lp-I, and Lp-I represent the faecal samples from mice at the beginning of animal experiment, while Control-F, FOS-F, FOS + Lp-F, and Lp-F represent the faecal samples from mice reared for 4 weeks (**D**,**E**). Different letters suggest a remarkable discrepancy according to *p* value (*p* < 0.05) between all groups.

To elucidate the reason behind the alteration in SCFA concentration by *L. plantarum* ST-III and FOS intervention, we analyzed the regulatory efficiencies of *L. plantarum* ST-III and FOS intervention on the gut microbiota structure using 16S rDNA high-throughput sequencing analysis. Three parameters of the alpha diversity index were measured for all groups. At the beginning of the experiment, there were no remarkably discrepancy in the Chao1, Simpson and Shannon indices of OTU levels among all groups (*p* > 0.05) (Figure S1). After 28 days of intervention, oral administration of *L. plantarum* ST-III remarkably decreased the Chao1 index relative to the control and FOS group (*p* < 0.05), but no statistical differences in Chao1 index between the standard diet-fed mice and FOS-fed mice (*p* > 0.05) (Figure 2A). Unexpected, long-time consumption of FOS significantly reduced the Simpson and Shannon index of intestinal microbiota compared with that of mouse fed a standard diet (*p* < 0.05) (Figure 2B,C). Among these, oral administration of *L. plantarum* ST-III remarkably decreased the Shannon index relative to the control or FOS groups (*p* < 0.05). Besides, *L. plantarum* ST-III intervention remarkably reduced the ratio of Firmicutes/Bacteroidetes relative to other groups (*p* < 0.05). Nevertheless, there were no remarkable discrepancies in the Firmicutes/Bacteroidetes rate among the control, FOS and FOS + Lp groups (*p* > 0.05) (Figure S2).

PCA were applied to deeply analyze the regulatory influence of *L. plantarum* ST-III and FOS intervention on the gut microbiota structure (Figure 2D,E). The results of PCA exhibited the PC1, PC2 and PC3 accounting for 15.06%,13.4% and 9.33% of the total variation, respectively. There were no obvious discrepancies in intestinal microbiota at the beginning of the experiment among all groups. After 4 weeks of intervention, fecal samples of the control-F and Lp-F groups phases in the PCA loading plot were scattered counterclockwise, while fecal samples of the FOS-F and FOS + Lp-F groups phases in the PCA loading plot were scattered clockwise (Figure 2D). Moreover, the intestinal microbiota of mice in the Lp-F group and that of the mice in the Control-F groups were quite different on principal component plots (PC1 vs. PC2). By contrast, no marked differences were observed between the FOS-F and FOS + Lp-F groups. As shown in Figure 2E (PC1 vs. PC3), the Control-F and FOS-F groups were the positive for PC3 (the second and first quadrants, respectively), while the Lp-F and FOD + Lp-F groups were the negative for PC3 (the third and fourth quadrants, respectively). In general, oral administration of *L. plantarum* ST-III regulated intestinal microbial structure in mice fed with a standard diet and/or FOS.

### 3.4. Influence of L. plantarum ST-III and FOS on the Abundance of Intestinal Microbiota

**Figure 3:**
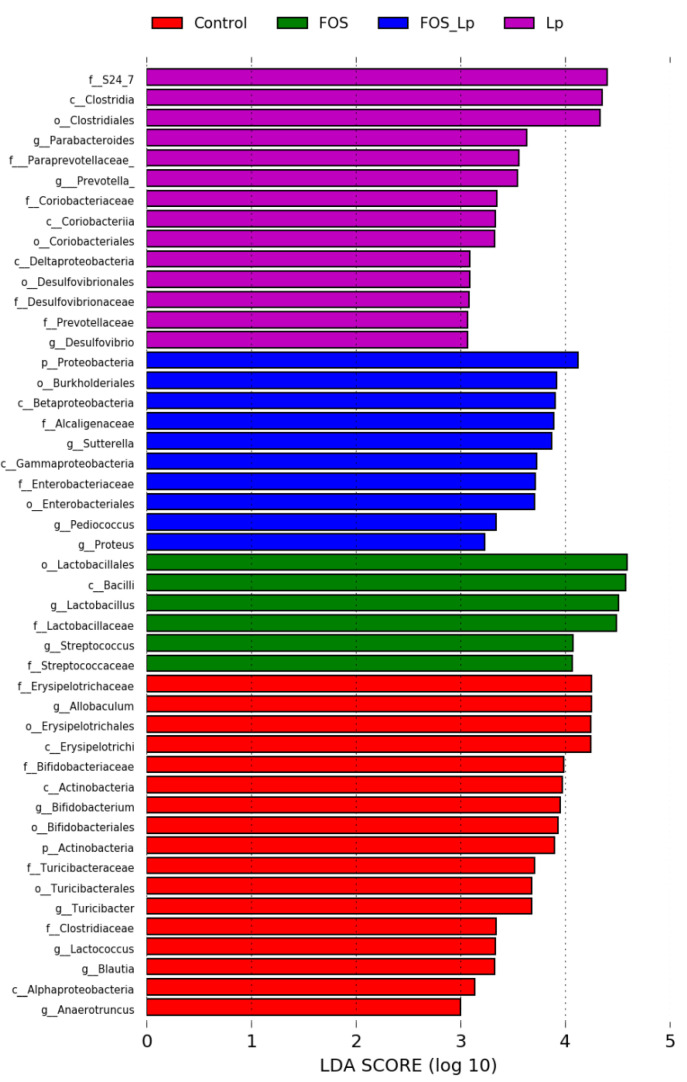
The LDA score is based on LEfSe analysis of the intestinal microbiota in the different groups.

To explore the changes of the proportion of intestinal microbial composition among the Control, FOS, Lp and FOS + Lp groups, the heatmap and linear discriminant analysis effect size (LEfSe) analysis were applied in the present study. As described in Figure S3, the intestinal microbiota structure was obviously shifted among the four groups. Linear discriminant analysis (LDA) based on (LEfSe) is applied to further reveal the representative composition of intestinal microbiota and their dominant bacteria (Figure 3). At the family level, long-time consumption of FOS increased the relative abundance of *Lactobacillaceae* and *Streptococcaceae*; FOS combined with *L. plantarum* ST-III intervention enhanced the relative abundance of *Alcaligenaceae* and *Enterobacteriaceae.* In addition, *L. plantarum* ST-III intervention elevated the proportion of S24-7, *Paraprevotellaceae*, *Coriobacteriaceae*, *Desulfovibrionaceae* and *Prevotellaceae*. At the genus level, long-term consumption of FOS enhanced the proportion of *Lactobacillus* and *Streptococcus*; FOS combined with *L. plantarum* ST-III intervention enhanced the proportion of *Sutterella*, *Pediococcus* and *Proteus*; *L. plantarum* ST-III intervention enhanced the proportion of *Parabacteroides*, *Prevotella* and *Desulfovibrio*. Collectively, these data suggest that FOS and/or *L. plantarum* ST-III interventions remarkably alter the intestinal microbiota structure.

### 3.5. Association between Intestinal Microbiota and SCFAs

**Figure 4:**
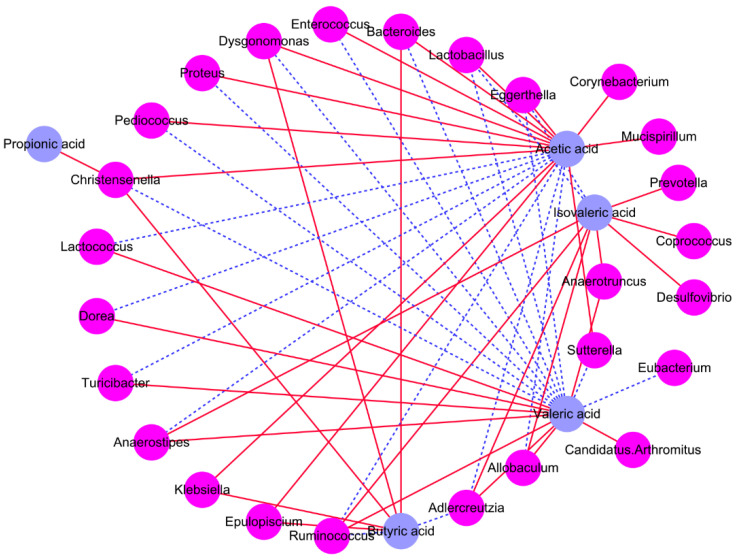
Correlation analysis between the specific compositional features of intestinal microbiota and the concentrations of cecal SCFAs.

To further explore the possible relationship between the levels of SCFAs and the dominant intestinal microbiota species, Spearman’s correlations were computed. The relative abundance of *Prevotella*, *Coprococcus*, *Anaerotruncus*, *Parabacteroides*, *Helicobacter*, *Atopobium*, *Desulfovibrio*, *Dehalobacterium*, *Ruminococcus*, *Adlercreutzia*, *Allobaculum, Turicibacter*, *Dorea*, *Anaerostipes*, *Lactococcus*, and *Candidatus*-*Arthromitus* were *positively* associated with the levels of valeric, propionic, and isovaleric acids, and are negatively related to the levels of acetic and butyric acids (Figure 4 and Figure S4). The relative abundance of *Epulopiscium*, *Dysgonomonas*, *Christensenella*, *Klebsiella*, *Corynebacterium*, *Pediococcus*, *Odoribacter*, *Mucispirillum* are positively related to the concentration of propionic, isovaleric, acetic and butyric acids, and are negatively associated with the concentrations of valeric acid. In addition, the relative abundance of *Sutterella*, *Proteus*, *Enterococcus*, *Eubacterium*, *Bacteroides*, *Lactobacillus* and *Eggerthella* are positively associated with the levels of valeric, propionic and isovaleric acids, and negatively related to the concentrations of acetic and butyric acids.

## 4. Discussion

Intestinal microbiota is responsible for the regulation of host energy metabolism. Clinical investigations elaborated that the imbalance in intestinal microbiota takes a crucial role in the pathological process of certain metabolic diseases, including nonalcoholic liver injury, hyperlipidemia, hyperglycemia, and cardiovascular diseases. Diet is one of the most important elements affecting the intestinal microbiota structure. FOS is generally regarded as a fermentable prebiotic, and the presence of FOS accelerates the proliferation of *Bifidobacterium* and *Lactobacillus* in gut. Among these, *L. plantarum* ST-III possesses the capacity to metabolize FOS by secreting β-fructosidase (SacA) and specific oligosaccharide transporters [9]. In addition, our previous results elaborated that *L. plantarum* ST-III intervention effectively enhanced the relative abundance of Lactobacillus, indicating that *L. plantarum* ST-III can reproduce and grow in the intestine [12]. However, the effects of daily consumption of FOS and *L. plantarum* ST-III on the intestinal microbiota structure in vivo have not been explored. In the present research, we found that FOS and/or *L. plantarum* ST-III intervention elevated the levels of SCFAs and changed the composition of the intestinal microbiota.

FOS is an important prebiotic that occurs naturally in fruits and vegetables. In this study, we found that FOS consumption effectively inhibits an increase in body weight, which is confirmed by previous study [13]. FOS enhances the number of defecations, decreases the hardness of feces, and excretes the volume of water [14]. In addition, we found that the moisture content of feces was significantly increased in the FOS group. However, oral administration of *L. plantarum* ST-III effectively inhibited a decrease of body weight, which is consistent with a previous report [15]. This may be because *L. plantarum* ST-III accelerates the fermentation of FOS and regulates the composition of intestinal microbiota [9]. In addition, FOS combined with *L. plantarum* ST-III intervention significantly enhanced the weight of ileum, cecum and colon. A previous report indicated that FOS ameliorates gastrointestinal conditions, promotes the absorption mineral, reduces serum lipid levels, and regulates the intestinal immune system [16]. In addition, the high abundance of *L. plantarum* ST-III provided nutrients for the proliferation of intestinal epithelial cells by accelerating the fermentation of indigestible FOS.

The beneficial effects of FOS and *L. plantarum* ST-III are considered as the result from increased *Lactobacillus* numbers and SCFAs levels in the gut. SCFAs are essential functional substances and are mainly stemmed from intestinal microbiota by their fermentation of indigestible carbohydrates. SCFAs offer energy to the gut microbiota and intestinal epithelial cells, and elevate the absorptivity of minerals, which effectively hamper the process of ulcerative colitis [17]. Some literatures suggested that certain intestinal bacteria synthesize SCFAs using dietary indigestible carbohydrates as substrates. First, the dietary indigestible carbohydrates are converted to monosaccharides through glycoside hydrolases, which are then fermented to SCFAs by the carbon metabolic pathways under anaerobic condition [18]. Many external factors, such as food composition, age, probiotics and faecal microbiota transplantation, influence cecal SCFAs concentration by regulating the intestinal microbiota structure [12]. Among these, food composition takes an essential role in affecting the concentrations of SCFAs in gut. Long-term macronutrient intervention helps maintain the fluctuations in the endogenous SCFAs concentrations. In this study, long-term consumption of FOS obviously increased the concentrations of cecal SCFAs relative to that of mice fed a standard diet, which may result from the FOS being converted into SCFAs through glycoside hydrolases originating from *Bifidobacterium* and *Lactobacillus* in the gastrointestinal tract [19]. However, oral administration of *L. plantarum* ST-III slightly reduced the levels of SCFAs in mice compared with the FOS group. This may be due to the fact that the cecal SCFAs act as a source of energy for the proliferation of *L. plantarum* ST-III, thus the concentration of SCFAs in the FOS + Lp group was lower than that in the FOS group. Acetic acid, a primary SCFA, may influence the biosynthesis of fatty acids and regulate leukocyte function [4]. An appropriate concentration of acetic acid helps maintains the integrity of the intestinal barrier by combining with the chemoattractant receptor GPR43 [4]. GPR43 is expressed in immune cells, and regulates the development of metabolism [20]. Propionic acid ameliorates intestinal inflammation by suppressing the transcription of LPS-stimulated cytokines and pro-inflammatory cytokines, and regulating neutrophil activation [21]. Butyric acid also takes a beneficial role in the amelioration of certain diseases through inhibiting the NF-κB-mediated NLRP3 pathway, and it also accelerates intestinal transit [22], which is an important cause for the loss of body weight of the mouse in the FOS group. However, previous research displayed that butyric acid promoted the proliferation of intestinal goblet cell, which may lead to the weight of cecal and colon [23]. In this study, oral administration of *L. plantarum* ST-III reduced the concentration of SCFAs in mice fed with FOS, possibly because *L. plantarum* ST-III major releases the butyric acid by the conversion of FOS. Generally, FOS and *L. plantarum* ST-III intervention significantly elevated the concentration of SCFAs, which led to an increase of the ileal, cecal and colon weight.

It is well accepted that the intestinal microbiota participates in the homeostasis of glycolipid metabolism and the immune system function. Food composition is a vital factor in the regulation of intestinal microbiota. Comprehending the association between food composition and intestinal microbiota in the context of human health is a critical problem in personalized nutrition [24]. On the one hand, active substances or prebiotics were extracted and separated from natural products and food alters the proportion of intestinal microbiota. On the other side, the intestinal microbiota regulates host health by secreting critical metabolites and signals. The appropriateness of this interaction is confirmed by few studies that show that the beneficial influence of biologically substances or prebiotic supplements in different clinical conditions is related to particular signatures of the intestinal microbiota [25]. In the present research, we found that FOS and *L. plantarum* ST-III interventions altered the intestinal microbiota structure. Chao1, Shannon, and Simpson indices of OTU levels are vital indexes in α-diversity analysis for assessing the diversity of species in the intestinal microbiota [25]. Our data displayed that *L. plantarum* ST-III and FOS interventions shifted these indices. Bacteroidetes and Firmicutes are the two primary phyla, and the ratio of Firmicutes to Bacteroidetes (F/B ratio) is strongly associated with host lipid metabolism. Increasing evidence has shown that the F/B ratio is commonly presented in individuals with obesity and mammals [26]. Oral administration of FOS and FOS + Lp did not shift the F/B ratio, whereas only the *L. plantarum* ST-III supplement remarkably reduced the F/B ratio, indicating that *L. plantarum* ST-III is beneficial in preventing the occurrence of obesity. From the perspective of the F/B ratio, the presence of FOS did not affect the host health conditions. At the genus level, our results revealed that the specific compositional features of intestinal microbiota differed among the control, FOS, Lp and FOS + Lp groups. *Lactobacillus* and *Streptococcus* form the predominant genera among the resident microbiota in the gut of mice in the FOS group. *Lactobacillus* stimulates innate and adaptive immune responses by combining a series of pattern recognition receptors in the immune cells and the intestinal epithelium, and thereby effectively strengthening the host immune function [27]. In addition, it has also been confirmed that the structure of *Lactobacillus* (including cell wall components, polypeptides and exopolysaccharides) plays a role in regulating immune response [28]. *Streptococcus* is a Gram-positive bacterium that prevents the development of chronic gastric diseases, especially colitis. *Streptococcus* strengthens the host immune system through irritating macrophages and lymphocytes [29]. *Parabacteroides* and *Desulfovibrio* are the predominant genera among the resident microbiota in the gut of mice in the Lp group. Previous study exhibited that the abundance of *Parabacteroides* was negatively associated with serum lipid profiles (including HOMA-IR, AUC, body weight gain, TC, TG and LDL-C), suggesting that higher abundance of *Parabacteroides* helps remove serum lipids and reduces the level of inflammation in the host [30]. In addition, we found that *L. plantarum* ST-III obviously increased the proportion of *Pediococcus* and *Proteus* in FOS-fed mice. The genus *Pediococcus* is composed of Gram-positive bacteria that produce high concentrations of acetic acid. In addition, previous study revealed that *Pediococcus* colonizes in the intestinal tract, and possesses the ability to eliminate serum TC and TG, and suppresses the release of pro-inflammatory cytokines [31]. These data suggest that FOS and *L. plantarum* ST-III treatment potentially accelerate the proliferation of beneficial bacteria in the host intestine.

## 5. Conclusions

In the present study, FOS and *L. plantarum* ST-III intervention elevated the concentrations of cecal SCFAs, especially for acetic, butyric, and valeric acids. In addition, FOS and *L. plantarum* ST-III effectively elevated the proportion of beneficial bacteria in the intestine. However, further research is necessary to explore the connection between the specific compositional features of the intestinal microbiota and the levels of SCFAs and inflammatory cytokines.

## Figures and Tables

**Table 1:** The cecal SCFAs concentrations were analyze by GC-MS.

	Control	FOS	Lp	FOS + Lp
Acetic acid (μmol/g)	10.92 ± 0.77 ^a^	35.23 ± 20.09 ^b^	12.35 ± 5.56 ^a^	31.63 ± 4.32 ^b^
Propionic acid (μmol/g)	3.05 ± 0.23 ^a^	7.15 ± 3.78 ^b^	2.91 ± 1.31 ^a^	5.04 ± 1.02 ^a^
Butyric acid (μmol/g)	0.96 ± 0.06 ^a^	8.05 ± 4.15 ^b^	1.38 ± 0.58 ^a^	10.53 ± 2.78 ^b^
Valeric acid (μmol/g)	0.77 ± 0.05 ^a^	2.91 ± 1.70 ^b^	0.80 ± 0.38 ^a^	1.42 ± 0.48 ^b^
Isovaleric acid (μmol/g)	0.70 ± 0.05 ^a^	1.70 ± 1.26 ^b^	1.24 ± 0.97 ^a,b^	0.89 ± 0.04 ^a,b^
SCFAs (μmol/g)	16.41 ± 1.10 ^a^	55.03 ± 30.92 ^b^	18.68 ± 8.80 ^a^	49.52 ± 7.83 ^b^

All data are described as means ± SD. Different letters suggest a remarkable discrepancy according to *p* value (*p* < 0.05) between all groups.

## Data Availability

The data showed in the present research are available on request from the corresponding author.

## Supplementary Materials

The following supporting materials can be downloaded at: https://www.mdpi.com/article/10.3390/foods11091187/s1. Table S1: The detailed composition of standard diet and FOS diet; Figure S1. Alpha and beta diversity of intestinal microbiota among all groups in the beginning of the experiment; Figure S2. Influence of *L. plantarum* ST-III and FOS intervention on the ratio of Firmicutes and Bacteroidetes; Figure S3. Influence of *L. plantarum* ST-III and FOS intervention on the relative abundance genera from each sample; Figure S4. Heatmap presenting the association between genera and cecal SCFAs. Blue means positive association and red means negative association.
