# Supplementary material for: Metagenomic Analysis of the Effects of Lactiplantibacillus plantarum and Fructooligosaccharides (FOS) on the Fecal Microbiota Structure in Mice
Source: Foods. 2022 Apr 19;11(9):1187. doi: 10.3390/foods11091187 (PMC9102988; doi:10.3390/foods11091187)
Supplement: Supplementary file 1 [file foods-11-01187-s001.zip › foods-1667125-supplementary.pdf]

Supplementary Materials

Supplementary Material 1：

Table S1. The detail composition of standard diet and FOS diet

Nutritional Components	Standard diet (g/1000g)	FOS diet (g/1000g)
Casein	200	197.33
Cornstarch	397.5	392.18
Dextrin	132	130.23
Soybean oil	70	80.86
Mineral mix	35	29.81
Vitamin mix	10	8.52
L-cystine	3.0	2.56
Choline chloride	2.5	2.13
Sucrose	150	6.38
FOS	0	150

Supplementary Material 2:

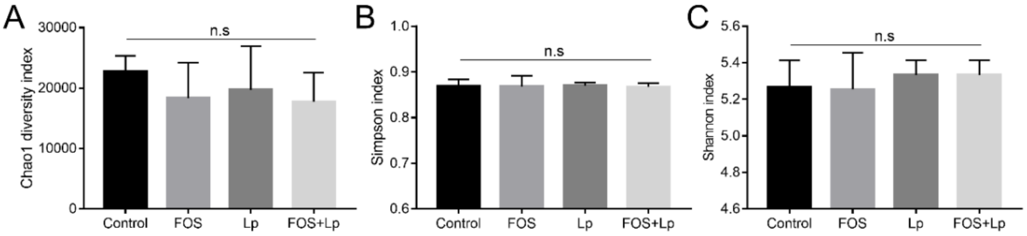

Figure S1. Alpha and beta diversity of intestinal microbiota among all group in the beginning of the experiment. Chao1 index (A), simpson index (B), and shannon index (C).

Supplementary Material 3：

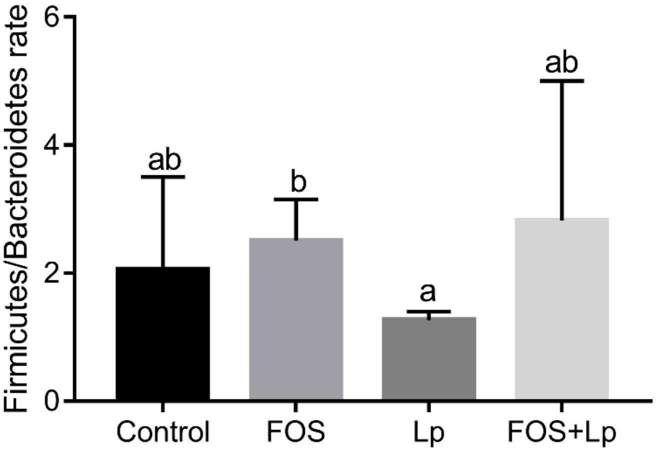

Figure S2. Influence of *L. plantarum* ST-III and FOS intervention on the ratio of Firmicutes and Bacteroidetes.

Supplementary Material 4:

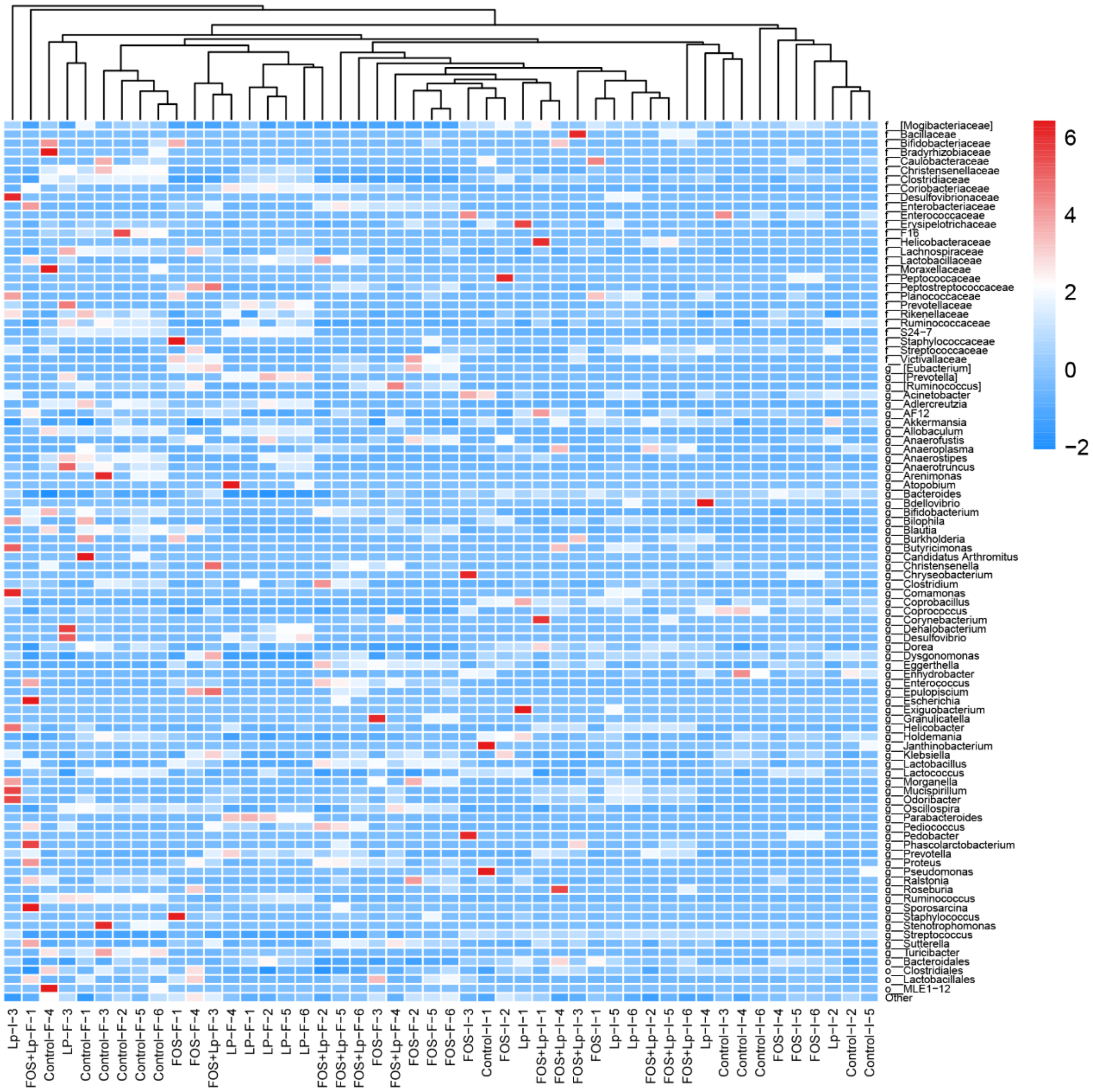

Figure S3. Influence of *L. plantarum* ST-III and FOS intervention on the relative abundance genera from each sample.

Supplementary Material 5:

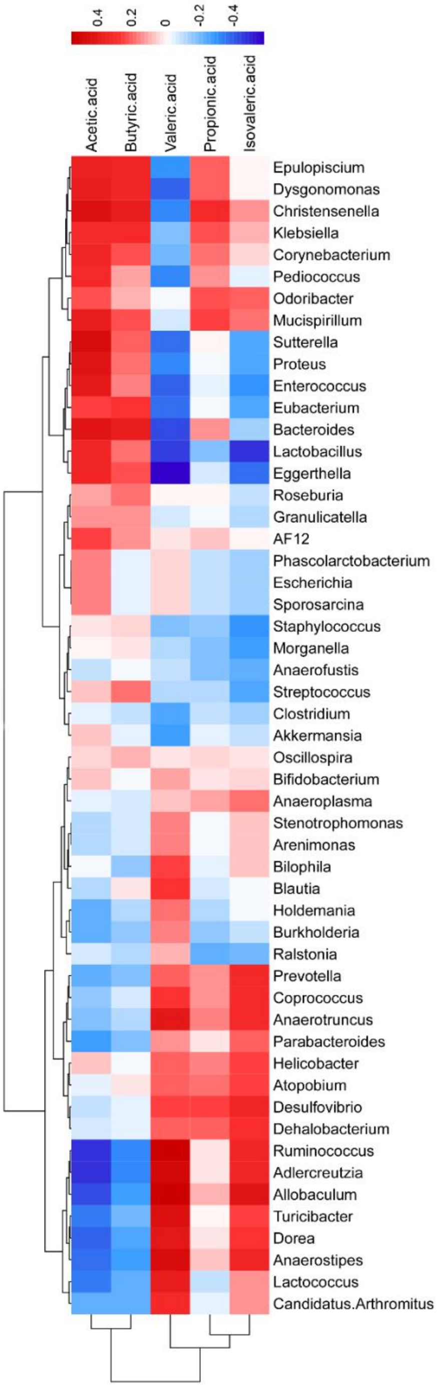

Figure S4. Heatmap presenting the association between genera and cecal SCFAs. Blue means positive association and red means negative association.
